# Variation in the metabolites and α-glucosidase inhibitory activity of *Cosmos caudatus* at different growth stages

**DOI:** 10.1186/s12906-019-2655-9

**Published:** 2019-09-05

**Authors:** Wan Ahmad Wan-Nadilah, Muhammad Tayyab Akhtar, Khozirah Shaari, Alfi Khatib, Azizah Abdul Hamid, Muhajir Hamid

**Affiliations:** 1 Faculty of Bioresources and Food Industry, Universiti Sultan Zainal Abidin, Besut, Terengganu Malaysia; 2 Laboratory of Natural Products, Institute of Bioscience, Universiti Putra Malaysia, Serdang, Selangor Malaysia; 3 Kuliyyah of Pharmacy, International Islamic University Malaysia, Kuantan, Pahang Malaysia; 4 Faculty of Food Science and Technology, Universiti Putra Malaysia, Serdang, Selangor Malaysia; 5 Faculty of Biotechnology and Biomolecular Sciences, Universiti Putra Malaysia, Serdang, Selangor Malaysia; 6 Institute of Industrial Biotechnology, Government College University, Lahore, Pakistan

**Keywords:** *Cosmos caudatus*, α-Glucosidase inhibition, Total phenolic content, Harvesting age, Quercetin derivatives, NMR-based metabolomics

## Abstract

**Background:**

*Cosmos caudatus* is an annual plant known for its medicinal value in treating several health conditions, such as high blood pressure, arthritis, and diabetes mellitus. The α-glucosidase inhibitory activity and total phenolic content of the leaf aqueous ethanolic extracts of the plant at different growth stages (6, 8. 10, 12 and 14 weeks) were determined in an effort to ascertain the best time to harvest the plant for maximum medicinal quality with respect to its glucose-lowering effects.

**Methods:**

The aqueous ethanolic leaf extracts of *C. caudatus* were characterized by NMR and LC-MS/MS. The total phenolic content and α-glucosidase inhibitory activity were evaluated by the Folin-Ciocalteu method and α-glucosidase inhibitory assay, respectively. The statistical significance of the results was evaluated using one-way ANOVA with Duncan’s post hoc test, and correlation among the different activities was performed by Pearson’s correlation test. NMR spectroscopy along with multivariate data analysis was used to identify the metabolites correlated with total phenolic content and α-glucosidase inhibitory activity of the *C. caudatus* leaf extracts.

**Results:**

It was found that the α-glucosidase inhibitory activity and total phenolic content of the optimized ethanol:water (80:20) leaf extract of the plant increased significantly as the plant matured, reaching a maximum at the 10th week. The IC_50_ value for α-glucosidase inhibitory activity (39.18 μg mL^− 1^) at the 10th week showed greater potency than the positive standard, quercetin (110.50 μg mL^− 1^). Through an ^1^H NMR-based metabolomics approach, the 10-week-old samples were shown to be correlated with a high total phenolic content and α-glucosidase inhibitory activity. From the partial least squares biplot, rutin and flavonoid glycosides, consisting of quercetin 3-*O*-arabinofuranoside, quercetin 3-*O*-rhamnoside, quercetin 3-*O*-glucoside, and quercetin 3-*O*-xyloside, were identified as the major bioactive metabolites. The metabolites were identified by NMR spectroscopy (*J*-resolve, HSQC and HMBC experiments) and further supported by dereplication via LC-MS/MS.

**Conclusion:**

For high phytomedicinal quality, the 10th week is recommended as the best time to harvest *C. caudatus* leaves with respect to its glucose lowering potential.

**Electronic supplementary material:**

The online version of this article (10.1186/s12906-019-2655-9) contains supplementary material, which is available to authorized users.

## Background

*Cosmos caudatus* belongs to the plant family Asteraceae. It is a popular local salad vegetable in Malaysia and is commonly known as “*ulam raja*”. Traditionally, young leafy shoots of the plant are recommended for the alleviation of diabetes, high blood pressure, arthritis and fever [1–3]. Other uses of the plant include a digestive aid and longevity [4, 5]. This plant has also been reported to have antioxidant and antidiabetic properties, which were concluded to be due to the presence of phenolic compounds [6–10].

Diabetes mellitus is a serious, debilitating disease that has become an increasing health burden to most of its sufferers. According to the International Diabetes Federation (IDF) [11], approximately 415 million people worldwide suffer from diabetes. This number is expected to rise to 642 million by 2040. The present trend indicates that more than 60% of the world’s diabetic population will be from Asia [12]. In Malaysia, an alarming 3.6 million adults older than 18 years are estimated to be affected by diabetes [13]. Currently, six classes of oral antidiabetic drugs comprising biguanides (e.g., metformin), sulfonylureas (e.g., glimepiride), meglitinides (e.g., repaglinide), thiazolidinediones (e.g., pioglitazone), dipeptidyl peptidase IV inhibitors (e.g., sitagliptin) and α-glucosidase inhibitors (e.g., acarbose, miglitol, voglibose and nateglinide) are in clinical use, either alone or together with insulin, to treat this disease [14, 15]. Unfortunately, these medications are also associated with various side effects and high secondary failure rates [14–16]. Therefore, there is an increased demand for safer and more effective alternative drugs or medicinal agents to treat or manage diabetes. Medicinal plants offer an interesting subject for further research into the identification of their bioactive constituents, their mechanism of action and their development into phytomedicinal products to help alleviate the disease.

The use of α-glucosidase inhibitors is a therapeutic approach used to reduce postprandial hyperglycaemia in diabetic patients. It works by retarding the absorption of carbohydrates and glucose [17]. The α-glucosidase enzyme is located in the brush border membrane of the small intestine, and its function is to breakdown carbohydrates to form the more absorbable monosaccharides. α-Glucosidase inhibitors delay and reduce postprandial glucose and insulin levels [18]. Thus, the inhibition of the carbohydrate-hydrolysing enzyme using α-glucosidase inhibitors is a plausible pathway to combat diabetes [19–21]. In recent years, numerous investigations have been carried out on the α-glucosidase inhibitory activity of various plant extracts in the hope of discovering new or more potent α-glucosidase inhibitors [22–26].

The quality of plant material produced from herb and medicinal plant plantations is greatly influenced by a number of factors, including the environment, cultivation conditions, and management practises, such as temperature, irradiance, fertilizer supply, irrigation and harvesting time [27, 28]. The cultivated plant material should be harvested at the optimum growth stage when the parameters associated with the raw material quality are at their peak [27, 29–32]. This is because different metabolites are biosynthesized and may be present at different stages of plant growth. The age of the crop at harvesting time is known to significantly affect the level of bioactive compounds in herbs and medicinal plants [31, 33, 34]. Thus, it is necessary to investigate the alterations in the production of the bioactive constituents in relation to the plant’s age to ascertain the optimum time for crop harvest that would assure the potency of the biological or pharmacological effect of the final plant product. For the herbal industry in particular, these aspects are very important in the standardization and quality control of the herbal produce, either in the upstream or the downstream stages.

Presently, study on variations in the levels of bioactive constituents with respect to the maturation of *C. caudatus*, specifically the α-glucosidase inhibitory properties, is still underexplored. Previously, Mediani et al. [29, 30] evaluated the antioxidant activity of *C. caudatus* at different growth stages along with the effect of various drying methods on the harvested sample. However, the influence of the growth stages on the α-glucosidase inhibitory activity of the harvested samples was not examined. Moreover, Javadi et al. [35] investigated the α-glucosidase inhibitory activity of *C. caudatus* samples, but this study specifically examined the effect of different post-harvest storage times (0–12 h). They reported that the biological activity decreased after 10 h of storage. In a continued study, Javadi et al. [36] further reported that some compounds contributing to the α-glucosidase activity were catechin, α-linolenic acid, α-D-glucopyranoside and vitamin E. Thus, more information is needed with regards to the best practices in the cultivation of this medicinal plant to provide proper guidelines and a standard operating procedure for farmers to produce quality plant material for a specific biological use.

The present study reports the evaluation of the metabolite profiles of *C. caudatus* at different stages of its growth and its correlation to the α-glucosidase inhibitory properties. The experiment was carried out with a specific cultivar and strictly followed agronomic conditions. The study was divided into two parts. First, to ensure good representation of the α-glucosidase inhibitory constituents, the best solvent for the optimum extraction of the bioactive compounds was determined. The optimum solvent system was then employed in the plant age-metabolite correlation study. An NMR-based metabolomics approach [37] was used to analyse the solvent-metabolite and plant age-metabolite correlations. Identification of the bioactive metabolites was further supported by 1D and 2D NMR spectroscopy and dereplication via LC-MS/MS metabolite profiling.

## Methods

### Chemicals

Absolute ethanol (EtOH) for the extraction of plant samples was purchased from HmBg Chemicals Inc. (Germany). For the α-glucosidase inhibitory assay, quercetin, glycine, 4-nitrophenyl-α-D-glucopyranoside and α-glucosidase from *Saccharomyces cerevisiae* were purchased from Sigma Aldrich (St. Louis, USA). Folin-Ciocalteu’s phenol reagent, gallic acid, and sodium carbonate (Na_2_CO_3_) were also supplied by Sigma Aldrich. For NMR measurements, deuterated methanol (CD_3_OD), non-deuterated KH_2_PO_4_, sodium deuterium oxide (NaOD), trimethylsilyl propionic acid-*d4* sodium salt (TSP) and deuterium oxide (D_2_O) were purchased from Merck (Darmstadt, Germany). For liquid chromatography mass spectrometry (LC-MS) analysis, HPLC grade methanol, acetonitrile, acetic acid and hydrochloric acid were purchased from Merck (Darmstadt, Germany). Liquid nitrogen was supplied by MOX Company (Petaling Jaya, Malaysia).

### Plant materials

Plant material used for the study was obtained from the germplasm collection of the Agriculture Technology Park, Universiti Putra Malaysia (UPM). Plant identity was confirmed by Dr. Shamsul Khamis, the botanist at the Institute of Bioscience, UPM, and a voucher specimen (No. SK 2511/14) was deposited at the Herbarium of the Institute. An experimental plot was established at the park in November 2013. Prior to planting, the soil was first treated, fertilized, turned and covered with black plastic to prevent weeds from growing in the soil. Four equidistant cut-out holes numbered 1 to 4 (thus groups of four per biological replicate) were made on the plastic covering, and 5 to 7 seeds were sown in the centre of each hole. As the plants grew larger, the number of plants was reduced to one per hole. Organic fertilizer was applied at the beginning of planting and every 2 weeks thereafter. Pesticide treatment was avoided during the growth period. Plants in the same plot were used for both solvent-metabolite and plant age-metabolite correlation metabolomics studies. For the solvent-metabolite correlation study, *C. caudatus* leaves were randomly collected from the plants growing in the plot and thoroughly mixed. Additionally, for the plant age-metabolite correlation study, leaf samples (comprising the top eight young leaves but excluding the shoot tip) were collected at different growth stages of the plant, i.e., at 6, 8, 10, 12 and 14 weeks. Each age group consisted of six individual plants (six biological replicates).

### Plant sample preparation

Leaf samples of *C. caudatus* were quenched directly by immersion in liquid nitrogen during field collection prior to transport to the laboratory for analysis. This was to ensure that the degradation of metabolites due to enzymatic reactions was minimized [38]. The samples were further freeze-dried before being ground into a fine powder. For the solvent-metabolite correlation study, the powdered leaf material was divided into 6 × 6100 g portions, for extraction with EtOH:water (E:W) solvent systems consisting of ratios of 100:0, 80:20, 60:40, 40:60, 20:80, and 0:100. To facilitate extraction, each replicate was sonicated for 1 h at room temperature. The extraction procedure for each replicate was repeated three times, and the filtrates were pooled, filtered (Whatman filter paper no 1), evaporated to dryness under vacuum, and stored at − 80 °C prior to metabolomics analysis. For the plant age-metabolite correlation study, the freeze-dried samples were similarly extracted using an 80:20 E:W solvent system. All samples were weighed, labelled and stored at − 80 °C prior to metabolomics analysis.

For ^1^H-NMR measurements, the sample preparation was carried out according to Kim et al. [39, 40] with slight modifications. A 10 mg sample of each extract was dissolved in 0.375 mL of deuterated methanol (CD_3_OD) and 0.375 mL of phosphate buffer (pH 6.0) prepared in D_2_O containing 0.1% TSP (w/w). The sample mixture was sonicated for 15 min, vortexed for 2–3 min and centrifuged at 13000 rpm for 10 min. The supernatant (0.6 mL) was collected, transferred to a 5 mm NMR tube and subjected to ^1^H-NMR measurement.

For LC-MS/MS analysis, each test sample was prepared by dissolving 1 mg of the extract in 1 mL of methanol and subjecting the solution to ultrasonication for 30 min at room temperature. The test sample was then filtered and kept at 4 °C prior to the analysis.

### Measurement of total phenolic content

The Folin-Ciocalteu method as described by Zhang et al. [41] was adopted for the measurement of total phenolic content (TPC), with minor modifications. Aliquots of 20 μL of each serial dilution (6.25, 12.5, 25, 50, 75, 100, 125, 250, 500 ppm) prepared from a stock solution (0.5 mg mL^− 1^) of the respective test extract were loaded onto a 96-well microplate, alongside the same series of serial dilutions of quercetin as a positive standard. Folin-Ciocalteu’s reagent (100 μL) was added to each well, mixed thoroughly using a vortex mixer, and the mixture was allowed to rest for 5 min at room temperature. This was followed by the addition of 80 μL of 7.5% (w/v) sodium carbonate solution and diluted to a final volume of 200 μL with distilled water. After thoroughly mixing, the plate was covered and left in the dark at room temperature. After 30 min, the absorbance of the reaction mixtures was measured at 765 nm against a blank (solvent used for extraction) using a microplate reader. The analysis was performed in triplicate. A standard calibration curve was constructed using gallic acid solutions of different concentrations (12.5, 25, 50, 75, 100, 125, 250, 500 and 1000 ppm). The TPC results are expressed as g gallic acid equivalents (GAE) per g dry weight of the fresh sample (g GAE g DW^− 1^).

### Measurement of α-glucosidase inhibitory activity

The α-glucosidase inhibitory activity was assayed in a 96-well plate following the method described by Collins et al. [42] with slight modifications. Briefly, 4 mg of plant extract was dissolved in 1 mL of ethanol to prepare a stock solution, and 5 mg mL^− 1^ quercetin in ethanol was used as a positive control [43]. Subsequently, 10 μL of sample solutions of different concentrations (0.003125, 0.00625, 0.0125, 0.025, 0.05, 0.1 and 0.2 mg mL^− 1^ for the test extract and 0.004, 0.008, 0.0156, 0.0313, 0.0625, 0.125, 0.25 mg mL^− 1^ for quercetin) were added to 100 μL of α-glucosidase type 1 from *S. cerevisiae* (Sigma G5003) solution (0.02 U well^− 1^) in 30 mM phosphate buffer (pH 6.5). The sample mixture was then incubated for 5 min at room temperature [44]. In the meantime, 60 mg of 4-nitrophenyl-α-D-glucopyranoside (PNPG) was dissolved in 20 mL of 50 mM phosphate buffer (pH 6.5). The PNPG solution (75 μL) was added to each well, and the reaction mixtures were incubated for an additional 15 min at room temperature. The reaction was terminated by adding 50 μL of 2 M glycine (pH 10) to each well. The optical densities (ODs) were then immediately read at 405 nm using a microplate reader [44]. The results are described as IC_50_ inhibition values. The percent inhibition was calculated using the following formula:

$$ \%\mathrm{inhibition}=\left[\right({\mathrm{A}}_{\mathrm{c}}-{\mathrm{A}}_{\mathrm{e}}/{\mathrm{A}}_{\mathrm{c}}\Big]\ \mathrm{x}\ 100\% $$

where A_c_ is the difference in absorbance between the control (with enzyme) and the blank control (without enzyme);and

A_e_ is the difference in absorbance between a sample (with enzyme) and the blank sample (without enzyme).

To calculate the IC_50_ value, a plot of the assay concentration (serial dilutions) of each test extract versus the % inhibition was constructed. The IC_50_ value was then estimated using the fitted line, y = mx + c (where m and c are numbers), and IC_50_ = (50-c)/m, where m is the slope of the line and c is the y-intercept.

### Measurement of NMR spectra

^1^H-NMR spectra were measured on a 500 MHz Varian UNITY INOVA NMR spectrometer (Varian Inc., California, USA) functioning at a frequency of 499.91 MHz and temperature of 26 °C. For data acquisition, a single pulse proton experiment with Presat was used with the following set of parameters (3.53 s acquisition time, 64 scans with 1.5 s presaturation delay, spectral width: − 2 to 14 ppm). For structural elucidation, both 1D and 2D NMR experiments were used. The *J-*resolved spectrum was acquired in 50 min 18 s, and the relaxation delay was 1.5 s. The heteronuclear multiple bond coherence (HMBC) spectra were obtained using 64 scans, which were achieved in 6 h, 9 min and 9 s.

### Multivariate data analysis

All the ^1^H-NMR spectra were manually phased and baseline corrected using Chenomx software (v. 5.1, Alberta, Canada). The ^1^H-NMR spectrum of each sample was processed and binned (0.04 ppm bin width) over the spectral region of δ 0.50 to δ 10.00 ppm. All spectra were then automatically converted to ASCII files and transferred into a Microsoft Excel (version 1997–2003) worksheet. Residual water (δ 4.70–4.90 ppm) and methanol (δ 3.27–3.31 ppm) regions were excluded from the spectral data to retain only signals from endogenous metabolites. The resulting dataset was saved as an Excel file and further subjected to multivariate analysis using SIMCA-P+ version 13.0 (Umetrics, Umea°, Sweden).

### LC-MS/MS analysis

For the LC-MS/MS analysis, a Dionex C18 reversed-phase column (Dionex, Sunnyvale, USA) with dimensions of 250 (l) × 2.0 mm (i.d.) and 2.5 μm particle size was used. Analysis was performed on a Dionex Ultimate 3000 HPLC equipped with a photodiode-array detector (PDA-3000) at 26.9 °C (thermostatted column compartment). The mobile phase used was double distilled water containing 0.1% acetic acid (solvent A) and HPLC grade acetonitrile containing 0.1% acetic acid (solvent B). The addition of acetic acid to the mobile phase enhanced compound peak sharpness by inducing the ionization of metabolites [45]. Sample elution was performed in a gradient manner with 10 to 100 mL for solvent A and 90 to 0 mL solvent B. The injection volume was 15 μL with a constant flow rate of 1.00 mL min^− 1^. The flow was split to allow 200 μL min^− 1^ of eluent into the mass spectrometer. The total LC run time was 35 min. The mass spectrometry (MS) measurement of the sample was performed on a MicroTOF mass spectrometer (Bruker Daltonik GmbH, Bremen, Germany). The source conditions were: nebulizer gas nitrogen (N_2_) at 0.2 bar and dry gas (N_2_) at 3.0 L min^− 1^, dry temperature at 180 °C, capillary voltage at 4500 V and end plate offset at − 500 V. Data acquisition was performed by HyStar Application version 3.2, while data processing was carried out with DataAnalysis Version 3.4 by Bruker Daltonik GmbH.

### Statistical analysis

The results are expressed as the mean ± SD. The statistical significance of the results was evaluated using one-way ANOVA with Duncan’s post hoc test. Significant differences were based on *p* values where *p* < 0.05 was considered significantly different. Correlations among the different activities were performed by Pearson’s correlation test, where the IC_50_ values were converted to 1/IC_50_ to inverse the relationship between absorbance and activity.

## Results and discussion

### Influence of solvent polarity on TPC and α-glucosidase inhibitory activity

The solubility of the different classes of chemical constituents in a plant’s metabolome varies based on the polarity of the solvent used for the extraction of the metabolites. To ensure that the plant’s natural therapeutic value is not lost, it is important to determine the best solvent for the optimum extraction of the bioactive chemical constituents of the plant. The main goal of extraction is to obtain as much of the secondary metabolites as possible in the sample. Thus, different solvent polarity systems are commonly tested to find the most suitable solvent for extraction purposes. The TPCs measured for various *C. caudatus* extracts using the different EtOH:water systems were thus investigated, and the results are shown in Table 1.

**Table 1:** Total phenolic content (TPC) and α-glucosidase inhibitory activity of different EtOH:water (E:W) systems of *C. caudatus*

E:W system	TPC (g GAE g DW^− 1^)	α-glucosidase inhibitory activity, IC_50_ (μg mL^− 1^)
100:0	0.80 ± 0.11^a^	69.88 ± 7.22^a^
80:20	0.79 ± 0.09^a^	39.18 ± 5.80^b^
60:40	0.26 ± 0.08^b^	113.56 ± 6.72^c^
40:60	0.18 ± 0.13^b^	nd
20:80	0.09 ± 0.03^b^	nd
0:100	0.24 ± 0.11^b^	nd
Quercetin	nd	110.50 ± 4.30^c^

Different letters in the column indicate significant differences (*p* < 0.05) using Duncan’s test. Values are expressed as the mean ± standard deviation (*n* = 3). *nd* not detected

The 100% EtOH extract showed the highest TPC value (0.80 g GAE g DW^− 1^), whereas the E:W (20:80) extract exhibited the lowest TPC (0.09 g GAE g DW^− 1^). The TPCs of the extracts decreased drastically with increasing water ratio. The TPC of the 100% water extract was significantly different (*p* < 0.05) than the TPCs obtained by using the 100% EtOH and E:W (80:20) solvent systems. However, the TPC of the 100% EtOH and E:W (80:20) extracts were not significantly different from each other. This indicated that the two solvent systems are similar in their efficiency to extract the phenolic compounds from *C. caudatus*. Although the TPC was slightly higher when only water was used as the extraction solvent (0.24 g GAE g DW^− 1^) compared to E:W (40:60 and 20:80), the differences were not significant.

The IC_50_ values were calculated for the 100% EtOH and E:W (80:20, 60:40) extracts and compared with quercetin (Table 1). The IC_50_ values of the extracts were significantly different (*p* < 0.05) from each other with the E:W (80:20) extract showing the highest inhibitory effect on α-glucosidase, with an IC_50_ value of 39.18 μg mL^− 1^. Moreover, the IC_50_ of the E:W (80:20) and 100% EtOH (69.88 μg mL^−^ 1) extracts were also significantly better than that of quercetin (110.50 μg mL^− 1^). The other extracts (E:W 40:60, 20:80 and 100% water) were considered inactive against the enzyme since they gave a low percent inhibition of the enzyme (Additional file 1: Table S1).

Previously, Ryu et al. [46] reported that the E:W solvent system has a greater ability to extract α-glucosidase inhibitors from plants rather than water alone as the extraction solvent. The results of the present study indicated that the more organic solvent present in the system, particularly E:W (80:20), the more efficient the extraction of α-glucosidase inhibitors from *C. caudatus*. This was not surprising since phenolic compounds are expected to be less soluble in aqueous systems due to the limited solubility of the phenyl group, the fact that they are non-polar in nature. Thus, the phenolic constituents in the plant extract were more soluble in the solvent systems containing more organic solvent than in the more aqueous solvent systems. The TPC results in the present study were in agreement with previous studies by Siddhuraju and Becker [47], Anwar et al. [48], Sultana et al. [49] and Wijekoon et al. [50]. Many other studies have reported on the effectiveness of organic solvents to extract plant metabolites with potent biological activities [47, 49, 51, 52]. Additionally, the use of different solvents of different polarities, such as hexane, dichloromethane (DCM), ethyl acetate (EtOAc) and butanol (BuOH), is important to obtain fractions with high free radical scavenging and α-glucosidase activities [53]. However, the most suitable solvents are aqueous mixtures containing EtOH, methanol (MeOH), acetone, and EtOAc [54]. Polar solvents are frequently used for recovering polyphenols from plant matrices. EtOH is a good solvent for polyphenol extraction and is also safe for human consumption. Based on these results, it was concluded that the extractability of the phenolic compounds in *C. caudatus* was influenced by the nature and polarity of the solvent system used in the extraction process. These findings were in good agreement with other reports made by similar studies on plant metabolites [55–57]. Based on these findings, the E:W (80:20) extract was selected for the extraction of *C. caudatus* for the plant age-metabolite correlation study.

### Correlation between TPC and α-glucosidase inhibitory activity

The relationships between the TPC and α-glucosidase inhibitory activity of the extracts were further evaluated using Pearson’s correlation analysis (Additional file 1: Table S2). The Pearson correlation coefficient (PCC) was applied to study the correlation between the TPC and α-glucosidase inhibitory activity of the extracts. The correlation between TPC and α-glucosidase inhibitory activity was significantly lower (r = 0.434, r = 0.407; *p* < 0.05). Although phenolic compounds may be involved and play an important role in the α-glucosidase inhibitory activity of the plant [46, 52], there may be other compounds in *C. caudatus* that could contribute to the α-glucosidase inhibitory activity in addition to the phenolics. Possible constituents could be terpenoids and other metabolites, such as glycosides, sugars and carbohydrates, which are major constituents in an aqueous plant extract [58, 59]. In fact, the carbohydrates in *C. caudatus* have been previously reported to act as competitive inhibitors of the enzyme α-glucosidase [60].

The findings of the present study led us to suggest that the bioactive compounds contributing to the α-glucosidase inhibitory of *C. caudatus* were mainly found in the polar or semi-polar extracts of the plant. This finding was consistent with other studies, such as Jung et al. [58], Wang et al. [61] and Wresdiyati et al. [62], who also reported that the significant α-glucosidase inhibitory activity in polar or semi-polar extracts was attributable to the presence of phenolics and flavonoids and their glycosides.

### NMR and LC-MS/MS analysis of the E:W (80:20) extract

**Fig. 1:**
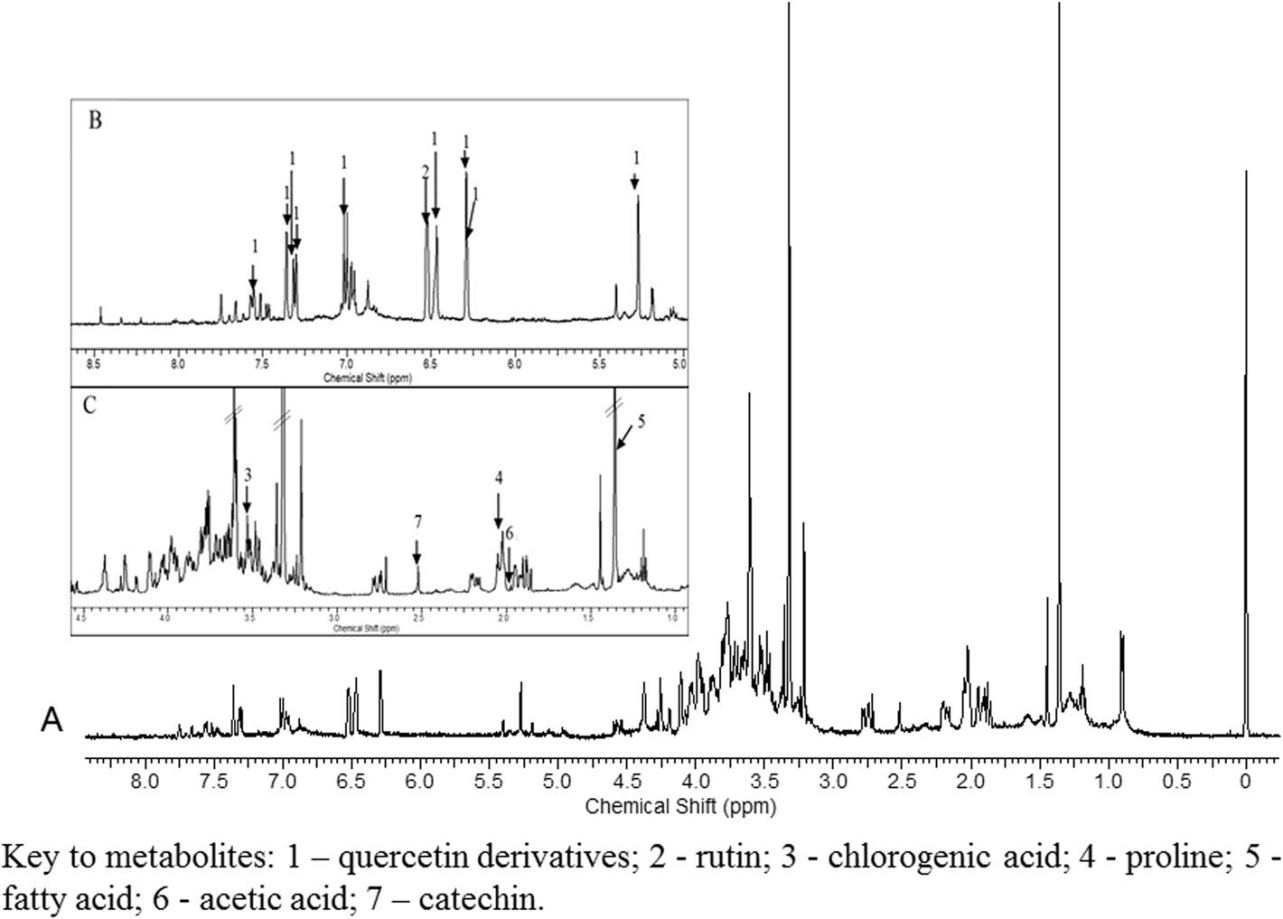
**a** Full ^1^H NMR spectrum of the EtOH:water (80:20) *C. caudatus* extract. **b** Expanded ^1^H NMR spectrum in the range of δ 5.0 to 8.5. **c** Expanded ^1^H NMR spectrum in the range of δ 1.0 to 4.5. Key to metabolites: 1 – quercetin derivatives; 2 - rutin; 3 - chlorogenic acid; 4 - proline; 5 - fatty acid; 6 - acetic acid; 7 – catechin

A representative ^1^H-NMR spectrum of the bioactive E:W (80:20) extract is shown in Fig. 1. Identification of the metabolites was based on comparison of the ^1^H-NMR chemical shifts with chemical shift values reported in the literature and further supported by the *J*-resolved and 2D experiments. The chemical shift assignments were deduced by matching the spin systems and *J* values compared to those reported in the literature. The ^1^H-NMR and ^13^C-NMR chemical shifts along with the *J* values are tabulated in Table 2. The *J*-resolved NMR spectra were measured to identify the overlapping peaks and to provide additional information on each signal (Additional file 1: Figure S1). Together with the *J*-resolved spectra, other 2D NMR correlation experiments such as HMBC, HSQC and COSY also facilitated the identification of the metabolites in the *C. caudatus* leaf extract.

**Table 2:** ^1^H NMR, ^13^C NMR characteristic signals and LC-MS/MS data of identified metabolites in *C. caudatus* extracts

Metabolite	NMR Chemical shift (ppm)		Molecular ion (M-H)^+^	MS/MS fragments
	δ_H_, mult (*J* in Hz)	δ_C_		
Quercetin	5.24, d (2)			
	5.28, d (7.8)			
	6.24, d (2)			
	6.28, d (2)			
	6.44, d (2)			
	7.02, d (8.5)			
	7.36, d (2)			
	7.28, dd (8, 2)			
	7.56, dd (8, 2)			
Quercetin 3-*O*-arabinofuranoside	6.28, d (2)		435.0780	303.0469, 181.0580, 153.9813
	7.02, d (8.5)			
	7.36, d (2)			
	7.56, dd (8, 2)			
	5.28, d (7.8)	111.37		
Quercetin 3-*O*- rhamnoside	6.28, d (2)		449.0954	303.0460, 181.0855, 153.0940
	7.02, d (8.5)			
	7.36, d (2)			
	7.56, dd (8, 2)			
	5.18, d (7.8)	95.72		
	0.90, d (1.1)	19.51		
Quercetin 3-*O*- glucoside	7.02, d (8.5)		465.0898	303.0466, 153.3540
	7.36, d (2)			
	7.56, dd (8, 2)			
	5.18, d (7.8)	95.43		
Quercetin 3-*O*-xyloside	6.28, d (2)		435.0801	303.0451, 181.6037, 153.8779
	7.02, d (8.5)			
	7.36, d (2)			
	7.56, dd (8, 2)			
	5.07, d (8.)	104.71		
Rutin	6.28, d (2)		611.1366	465.0893, 303.0466
	7.02, d (8.5)			
	7.36, d (2)			
	7.56, dd (8, 2)			
	4.99, d (7.5)	105.01		
	3.78, d (1.5)	65.20		
	4.39, d (1.5)	101.93		
	0.92, d (6.6)	19.06		
Chlorogenic acid	3.52, dd (10, 4)			
Fatty acid	1.32, m			
Acetic acid	1.92, s			
Catechin	2.56, dd (16, 7.5)			

The ^1^H NMR spectrum of the *C. caudatus* extract can be divided into three different regions consisting of the aromatic (δ 8.00 – δ 5.50), sugar (δ 5.50 – δ 3.00) and organic acid (δ 3.00 – δ 0.00) regions. Major metabolites, including an organic acid (i.e., acetic acid), an amino acid (i.e., proline), a fatty acid and phenolic compounds (i.e., quercetin derivatives, rutin, catechin, and chlorogenic acid) were identified. The visual inspection of ^1^H-NMR showed higher intensities of the identified peaks in the 10-week-old sample compared to the samples of the other age groups, especially the 12-week-old sample (Additional file 1: Figure S2). Since there were clear chemical shift differences observed in the aromatic and sugar regions of the ^1^H-NMR spectra at the different age groups (Additional file 1: Figure S2), further detailed analysis focused on these regions in their respective *J*-resolved spectra.

In the aliphatic region (δ 0.5–3.0), the amino acid was identified as proline at δ 2.04 (dd) *J* = 8.6, 6.4 Hz. Additionally, fatty acid signals were detected at δ 1.32 (m), and the organic acid at δ 1.92 (s) was successfully identified as acetic acid. Despite severe overlap, the signals of chlorogenic acid and catechin were also identified at δ 3.52 (dd) *J* = 10.0 Hz, 4.0 Hz and δ 2.56 (dd) *J* = 7.5 Hz, 16.0 Hz, respectively. In the aromatic region (δ 5.5–8.0), the signals were assigned to rutin and quercetin derivatives. The quercetin derivatives were assigned based on the characteristic signals at δ 5.24 (d) *J* = 2.0 Hz, δ 5.28 (d) *J* = 7.8 Hz, δ 6.24 (d) *J* = 2.0 Hz, δ 6.28 (d) *J* = 2.0 Hz, δ 6.44 (d) *J* = 2.0 Hz, δ 7.02 (d) *J* = 8.5 Hz, δ 7.36 (d) *J* = 2.0 Hz, δ 7.28 (dd) *J* = 8.0 Hz, 2.0 Hz, and δ 7.56 (dd) *J* = 8.0 Hz, 2.0 Hz. Rutin was detected at δ 6.52 (d) *J* = 2.0 Hz. The signals for the anomeric protons of the arabinosyl, rhamnosyl, glucosyl and xylosyl moieties were found at δ 5.28 (d) *J* = 7.8 Hz*,* δ 0.90 (d) *J* = 1.1 Hz, δ 5.18 (d) *J* = 7.8 Hz and δ 5.07 (d) *J* = 8.0 Hz, respectively. Furthermore, Table 2 shows the ^1^H-NMR and ^13^C-NMR characteristic signals of quercetin, quercetin derivatives, rutin, chlorogenic acid, proline, the fatty acid, acetic acid and catechin that were identified in this study.

To confirm the NMR data, the E:W (80:20) leaf extract was also analysed by LC-MS/MS. Further confirmation of the metabolites was based on the molecular ion peaks and MS/MS fragmentation of the parent compound (Table 2). The metabolites identified from LC-MS/MS were rutin, quercetin 3-*O*-glucoside, quercetin 3-*O*-xyloside, quercetin 3-*O*-arabinofuranoside and quercetin 3-*O*-rhamnoside (Additional file 1: Figure S3). The mass spectral data generated in this study were compared with the MassBank Database (High Quality Mass Spectral Database) and were found to be in good agreement with previous reports on *C. caudatus* [2, 6, 29, 30, 63].

### Multivariate data analysis of different *C. caudatus* extracts

**Fig. 2:**
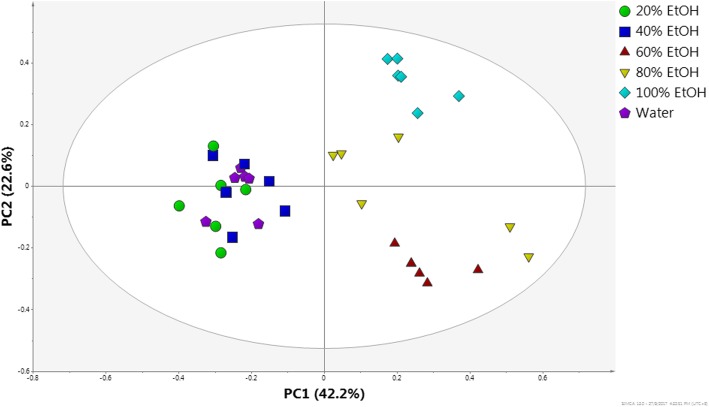
PCA score plot of the ^1^H NMR data of the various EtOH:water extracts

**Fig. 3:**
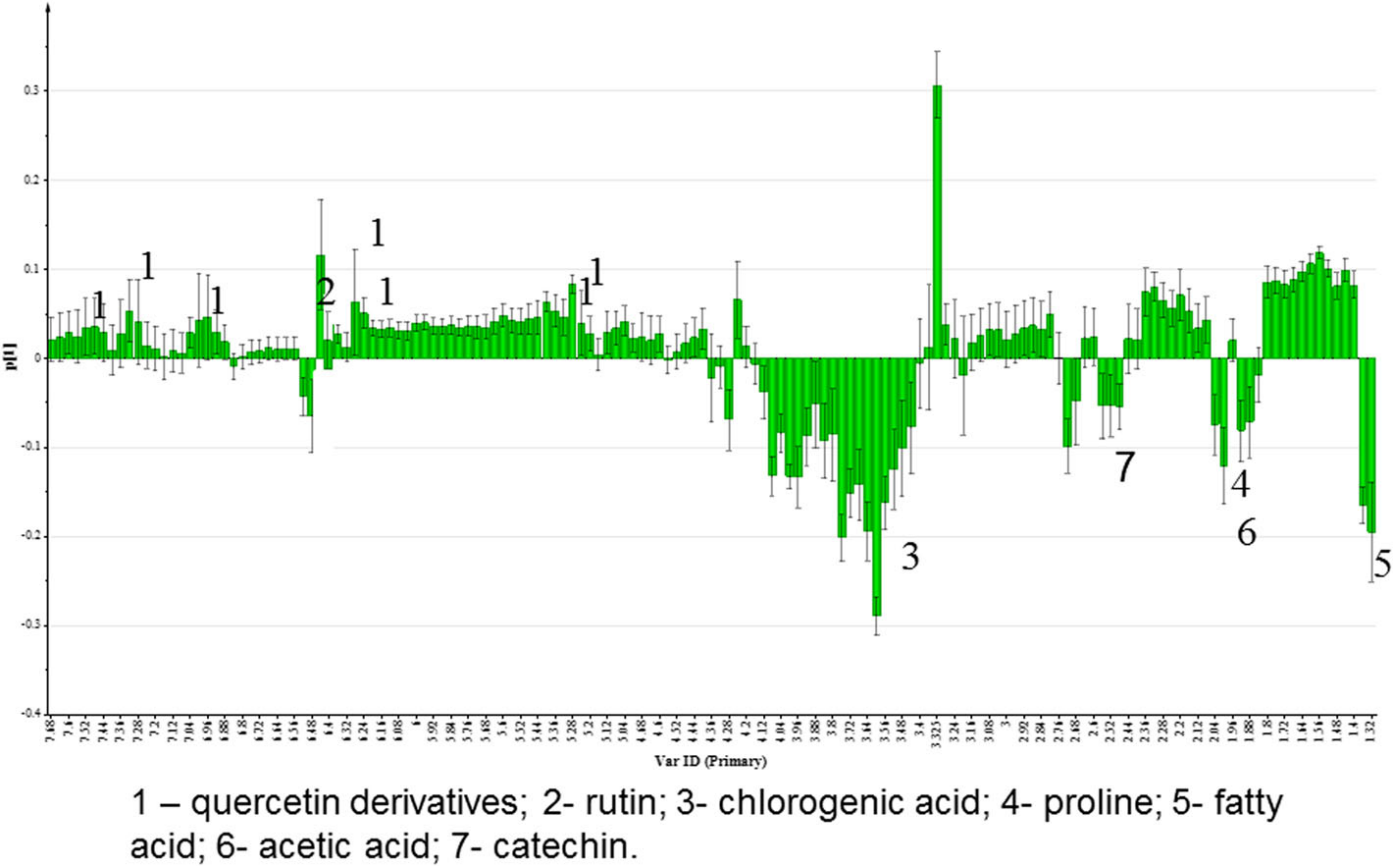
PCA loading column plot of the ^1^H NMR data of the various EtOH:water extracts. 1 – quercetin derivatives; 2 - rutin; 3 - chlorogenic acid; 4 - proline; 5 - fatty acid; 6 - acetic acid; 7 - catechin

Principal component analysis (PCA) was performed on the ^1^H-NMR data of the *C. caudatus* extracts. The PCs are illustrated graphically as score and loading plots, which are used to visualize the differences or similarities among the samples [64]. The PCA score plot showed a clear separation between the *C. caudatus* samples extracted with different solvent polarities (Fig. 2). The extracts obtained from the systems containing more organic solvent (E:W 80:20, 60:40 and 100% EtOH) were clustered together on the positive side of PC1, whereas the extracts obtained from the more aqueous solvent systems (E:W 40:60, 20:80 and 100% water) were grouped together on the negative side of PC1. PC1 accounted for 40.0% of the variation (Fig. 2), while PC2 accounted for 23.5%, amounting to an eigenvalue of 63.5%. From the loading column plot (Fig. 3), it was observed that the chemical shifts (δ 5.24, δ 5.28, δ 6.24, δ 6.28, δ 6.44, δ 7.02, δ 7.36, δ 7.28 and δ 7.56) attributable to the quercetin derivatives and rutin (δ 6.52) were the main contributors to the separation between the two groups of extracts in the PCA score plot.

**Fig. 4:**
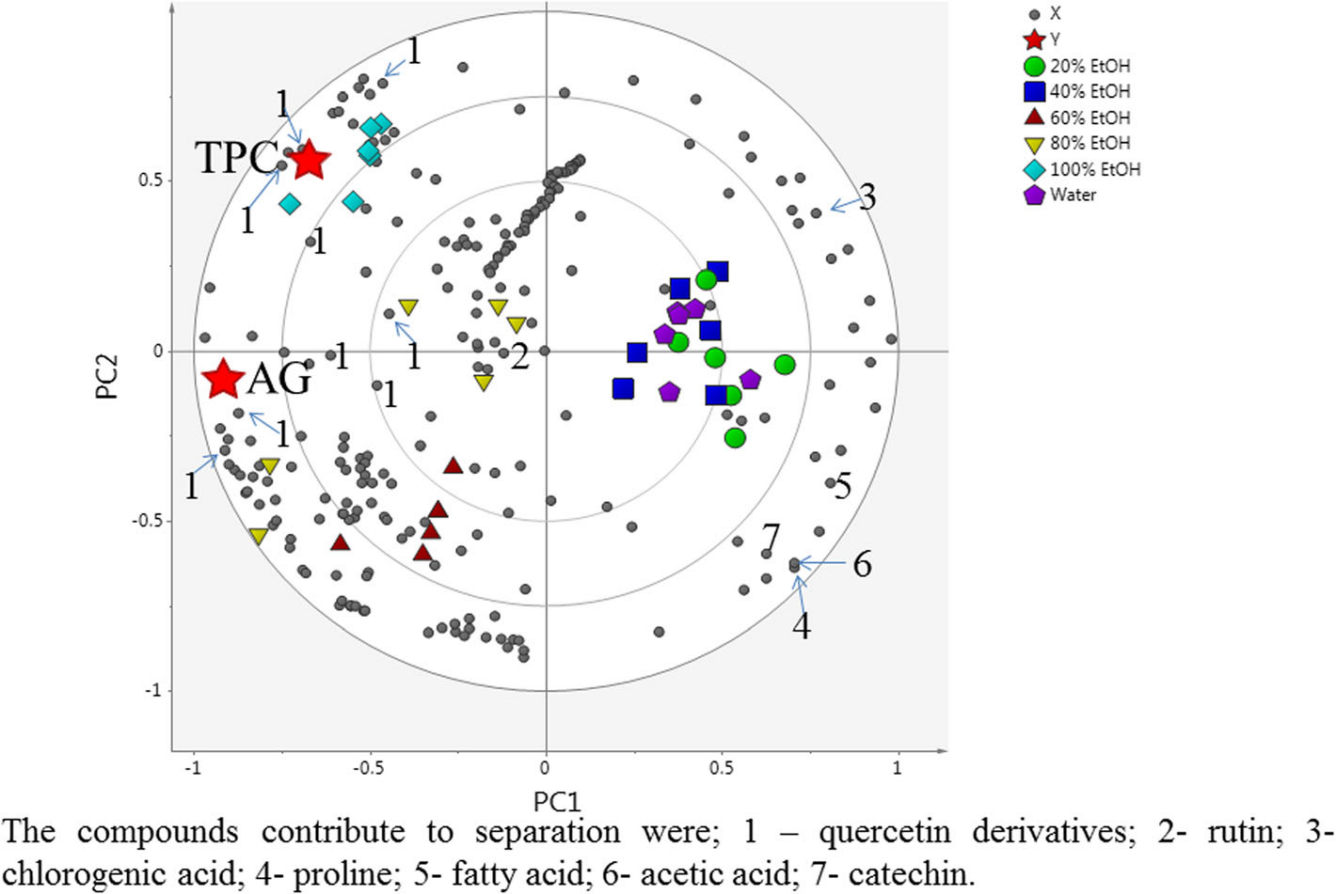
The biplot obtained from PLS describing the variations between the different EtOH:water extracts of *C. caudatus* and the correlation between the metabolites in the extracts with TPC and α-glucosidase inhibitory activity. The following compounds contribute to the separation: 1 – quercetin derivatives; 2 - rutin; 3 - chlorogenic acid; 4 - proline; 5 - fatty acid; 6 - acetic acid; 7 - catechin

Partial least squares (PLS) analysis was further performed to understand the correlation between the α-glucosidase inhibitory activity and the metabolites in the *C. caudatus* extracts. In the PLS biplot (Fig. 4), the E:W (80:20) extract showed a more pronounced correlation with the high TPC and the α-glucosidase inhibitory activity. The variable importance in projections (VIP) values, which helped to indicate the main components that contributed to the biological activity, were also determined using PLS-derived score plots (Additional file 1: Table S3). In general, the variables with VIP > 0.7 [65] were considered to significantly contribute to the separation of the groups observed in the PLS model. The VIP values of the major compounds contributing to the α-glucosidase inhibitory activity are given in the additional file (Additional file 1: Table S3). The metabolites that correlated with high TPC and α-glucosidase activity were mainly rutin and the quercetin derivatives. Previous reports have also shown that flavonoids have a significant correlation with the antidiabetic activity and antioxidant properties of many plants [66–69].

### Variation of TPC and α-glucosidase inhibitory activity at different stages of growth

The TPC measured for the E:W (80:20) extracts of *C. caudatus* harvested at different stages of growth are shown in Table 3. The extract of the 10-week-old sample showed the highest TPC (0.47 g GAE g^− 1^) and was significantly different from the rest of the samples, which varied between 0.24 and 0.37 g GAE g^− 1^. These results were consistent with the high TPC in the growth period preceding the flowering stage (10th week), followed by a distinct drop in the content after flowering (12- and 14-week-old). The IC_50_ values of the 8- to 14-week-old samples (24.11 to 78.90 μg mL^− 1^) were all significantly better than quercetin (110.5 μg mL^− 1^), indicating that *C. caudatus* contained more potent inhibitors of this enzyme. Based on the IC_50_ value recorded for the 10-week sample, it was proposed that the best age to harvest the leaves of *C. caudatus* is at the 10th week with respect to a high TPC and α-glucosidase inhibitory activity. The 6-week-old sample exhibited very low inhibition of the enzyme (Additional file 1: Table S4).

**Table 3:** Total phenolic content (TPC) and α-glucosidase inhibitory activity from different harvesting ages of *C. caudatus* extracts

Harvesting age (weeks old)	TPC (g GAE g DW^−1^)	α-glucosidase inhibitory activity, IC_50_ (μg mL^−1^)
6	0.24 ± 0.07^a^	nd
8	0.25 ± 0.03^a^	78.90 ± 0.01^a^
10	0.47 ± 0.05^b^	24.11 ± 0.03^b^
12	0.28 ± 0.04^a^	30.50 ± 0.01^c^
14	0.34 ± 0.05^a^	31.83 ± 0.04^c^
Quercetin	nd	110.50 ± 4.30^d^

Different letters in a column indicate significant differences (*p* < 0.05) using Duncan’s test. Values are expressed as the mean ± standard deviation (n = 3). *nd* not detected

The crucial factor in assuring the potency of the biological or pharmacological effects of the final plant extract is the harvesting age. This is because different metabolites are biosynthesized and may be present at different stages of the plant’s growth. It has also been shown that plants from different harvesting ages exhibited different bioactivities. Thus, the harvesting time should be optimized to preserve the plant’s health benefits and therapeutic properties of the final product. Determination of the appropriate growth stage for harvesting has provided valuable information on a plant’s nutritional and bioactivity properties [27, 29, 30]. It is interesting to note that *C. caudatus* extracted in EtOH:water (80:20) and harvested at the 10th week possess remarkable phenolic content and α-glucosidase inhibitory activity. The results were similar to those reported by Mediani et al. [30] and Siddiqui et al. [31], who also reported that the phenolic contents in *C. chinenses cv. Habanero* samples decreased with plant maturity. These changes may be caused by various factors, such as developmental stages [33, 34] and environmental conditions [32]. The study on *Ipomoea aquatica* by Lawal et al. [27] revealed that the phenolic content of a plant increases as the plant grows older. Similar results were reported for the leaves of *Moringa oleifera* [70], in that a higher total phenolic content and antioxidant capacity were detected in the mature leaves compared to the young leaves. Likewise, the levels of chlorophyll and carotenoids in the leaves of *Zea mays* were found to be dependent on the plant’s age [71]. Variation was also detected in the phytochemical contents of Brassica vegetables based on their developmental stages [72]. However, the study on *Averrhoa carambola* L. showed the reverse trend for antioxidant activity as the ripening process progressed [28].

### Multivariate data analysis of the *C. caudatus* extract obtained at different harvesting ages

**Fig. 5:**
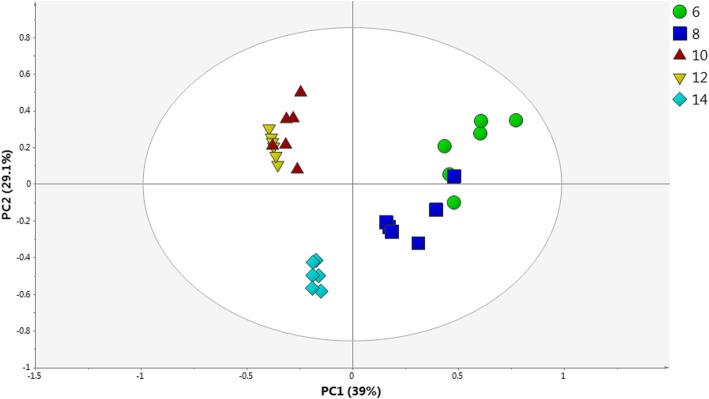
PCA score plot of the ^1^H NMR data of different harvesting ages of *C. caudatus* extracts

**Fig. 6:**
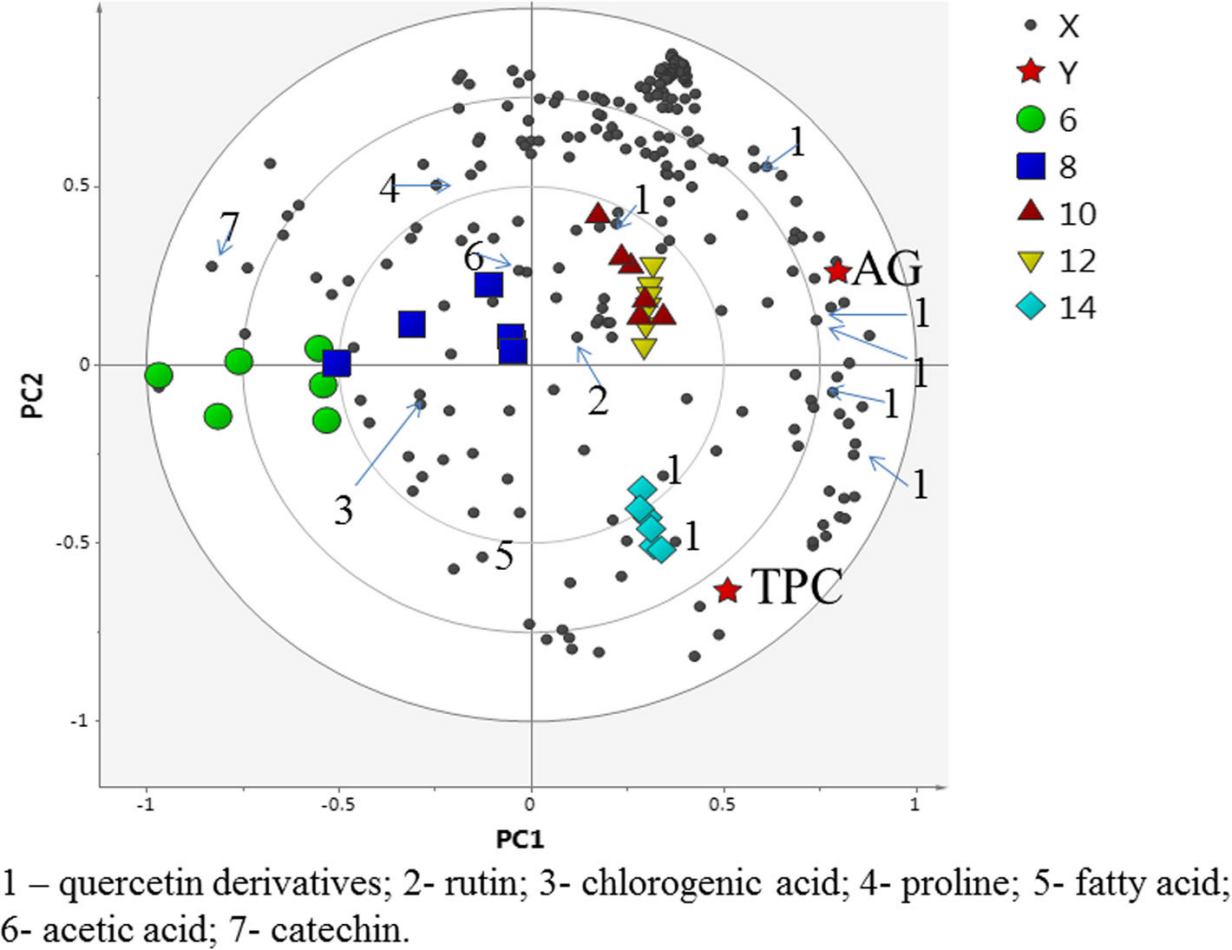
The biplot obtained from PLS describing the variations between the different harvesting ages of *C. caudatus* and the correlation between the metabolites in the extracts with TPC and α-glucosidase inhibitory activity. The following compounds contribute to the separation. 1 – quercetin derivatives; 2 - rutin; 3 - chlorogenic acid; 4 - proline; 5 - fatty acid; 6 - acetic acid; 7 – catechin

The PCA score plot (Fig. 5) showed a clear separation of the 10, 12 and 14 week samples from the 6 and 8 week samples. The 6- and 8-week-old samples were both projected on the positive side of PC1, while the 10-, 12- and 14-week-old samples were found on the negative side of PC1. However, the 14-week-old samples were also differentiated from the 10- and 12-week-old samples on the basis of PC2, with the week 14 sample having a negative PC2 score. The loading column plot showed that the chemical shifts indicative of the quercetin derivatives and rutin on the negative side of PC1 corresponded to the 10-, 12- and 14-week-old samples (data not shown). Moreover, on the positive side of the PC1 loading plot, chemical shifts attributable to the fatty acid, chlorogenic acid, proline, acetic acid and catechin were higher in the 6- and 8-week-old samples. In the PLS biplot (Fig. 6), the 10-, 12- and 14-week-old samples were well correlated with high TPC and α-glucosidase inhibitory activity. Again, the metabolites correlated with high TPC and α-glucosidase activity were mainly rutin and quercetin derivatives. The chemical shifts for the quercetin derivatives and rutin were located closer to the 10-, 12- and 14-week-old samples. Thus, the biological activities of the 10-, 12- and 14-week-old extracts were related to the presence of the phenolic components. Correlations to the TPCs further suggested that these metabolites were the major constituents in the bioactive extracts. Additionally, PLS analysis showed a strong correlation between the 10-, 12- and 14-week-old samples with α-glucosidase inhibition activity as well as phenolic contents, which formed the basis of the conclusion that the best quality *C. caudatus* material is obtainable after 10 weeks of growth. The VIP values of the major compounds contributing to the α-glucosidase inhibitory are given in Additional file 1: Table S3.

### Relative quantification of metabolites

Metabolite identity and concentration are possible discriminants of variation between the extracts. Therefore, the relative quantification of the metabolites in the different *C. caudatus* samples at different harvesting ages was also evaluated using GraphPad Prism software. The relative quantifications of the key metabolites are shown in Additional file 1: Figure S4.

In the present study, fatty acids were present in high amounts in young leaves (i.e., 6 and 8 weeks). The fatty acid levels as well as the degree of saturation were different at the different ages of the leaves. According to Ketchi and Kuiper [73], young leaves showed a higher concentration of fatty acids in the leaves of plants and these levels decreased as the growth period increased. Furthermore, as shown in Additional file 1: Figure S4, the fatty acid content increased in 12-week-old plants (flowering stages). This result was in agreement with a previous study by Nguyen et al. [74], which showed that rapid fatty acid accumulation started in the early stages of flowering. Saturated and polyunsaturated acids were the dominant fatty acids at this stage but their levels decreased after this date. These results were similar to the VIP value of fatty acid, which is ranks first but gives a negative contribution to the bioactivity of these samples.

Moreover, the level of catechin was also reported to be higher in young leaves, but no significant differences (*p* > 0.05) were reported among the plants of different ages them. According to Ghasemzadeh et al. [75], a decrease in the concentration of catechin in the leaves of *Zingiber officinale* Roscoe from 8 to 16 weeks after planting was observed. This finding was consistent with the results in Additional file 1: Figure S4, which showed a low concentration of catechin in 10-, 12- and 14-week-old samples.

As shown in Additional file 1: Figure S4, a higher concentration of quercetin derivatives, including rutin, was detected in the 10-, 12- and 14-week-old samples in comparison to the young leaf samples at 6 and 8 weeks old although no significant differences (p > 0.05) were reported between the groups. The 10-week-old extract showed a higher concentration of quercetin derivatives, including rutin, compared to the other extracts. Lower concentrations of chlorogenic acid, proline, fatty acid, acetic acid and catechin were observed in the 10-week sample.

Consequently, this plant can be suggested as a potential natural source of antioxidant and antidiabetic compounds for the prevention or treatment of diabetes and its complications. However, the use of this plant as an alternative remedy for diabetes requires more extensive studies, isolation of the bioactive compounds and safety and efficacy evaluations on human subjects.

## Conclusions

In the present study, α-glucosidase inhibitory activity data of *C. caudatus* leaves at different growth stages extracted with different solvent combinations were correlated with NMR results. Among the six EtOH:water solvent combinations, *C. caudatus* leaves extracted with EtOH:water (80:20) showed the highest α-glucosidase inhibitory activity. Among the harvesting ages, 10-week-old *C. caudatus* leaf samples were found to be the best in terms of α-glucosidase inhibitory activity. Rutin, quercetin 3-*O*-glucoside, quercetin 3-*O*-xyloside, quercetin 3-*O*-arabinofuranoside and quercetin 3-*O*-rhamnoside were identified as the major flavonoid glycosides responsible for the plant’s activity. Moreover, through ^1^H-NMR metabolomics along with multivariate data analysis, a strong correlation was found between TPC and *C. caudatus* leaf samples harvested at the 10th week and extracted with EtOH:water (80:20). Our results indicated the best age and solvent combination to harvest and extract *C. caudatus* leaves, respectively, to retrieve the bioactive constituents with maximum α-glucosidase inhibitory activity.

## Additional files

Additional file 1: **Table S1.** Maximum percent inhibition of α-glucosidase activity of different EtOH:water (E:W) extracts of *C. caudatus*. **Table S2.** Pearson’s correlation coefficient (r) between TPC and α-glucosidase activity of the EtOH:water extract solvent system at different harvesting ages. **Table S3.** VIP values of the major contributing compounds in the PLS model. **Table S4**. Maximum percent inhibition of α-glucosidase activity of the *C. caudatus* E:W (80:20) extract at different harvesting ages. **Figure S1.** Full spectrum of a representative *J*-resolved spectrum of an EtOH:water (80:20) *C. caudatus* extract from δ 0.0 to 8.0. Key to metabolites: 1 – quercetin derivatives; 2 – rutin; 3 - chlorogenic acid; 4 - proline; 5 - fatty acid; 6 - acetic acid; 7- catechin. **Figure S2.** (A) Full ^1^H NMR spectra of 6-, 8-, 10-, 12- and 14-week-old samples of *C. caudatus*. (B) Expanded ^1^H NMR spectra of the samples in the range of δ 5.5 to 8.5. Samples were dissolved in CH_3_OH-*d*4-KH_2_PO_4_ buffer in D_2_O (pH 6.0). Key to metabolites: 1 – quercetin derivatives; 2 - rutin; 3 - chlorogenic acid; 4 - proline; 5 - fatty acid; 6 - acetic acid; 7 – catechin. **Figure S3.** Positive ion mode MS/MS spectra of peaks 1 to 5. Peak 1, rutin; peak 2, quercetin 3-*O*-glucoside; peak 3, quercetin 3-*O*-xyloside; peak 4, quercetin 3-*O*-arabinofuranoside and peak 5, quercetin 3-*O*-rhamnoside. **Figure S4.** Relative quantification of metabolites in 6-, 8-, 10-, 12- and 14-week-old samples of *C. caudatus* at different harvesting ages. An * indicates significant differences (*p* < 0.05), ** indicates (*p* < 0.01) and *** indicate (*p* < 0.001) compared to the 6-week-old sample. Values are expressed as the mean ± standard deviation (*n* = 6). (DOCX 376 kb)

## Data Availability

All data and materials are described within the article and its supplementary information files.
