# Supplementary material for: Variation in the metabolites and α-glucosidase inhibitory activity of Cosmos caudatus at different growth stages
Source: BMC Complement Altern Med. 2019 Sep 5;19:245. doi: 10.1186/s12906-019-2655-9 (PMC6727405; doi:10.1186/s12906-019-2655-9)
Supplement: Supplementary file 1 — Table S1. Maximum percent inhibition of α-glucosidase activity of different EtOH:water (E:W) extracts of C. caudatus. Table S2. Pearson’s correlation coefficient (r) between TPC and α-glucosidase activity of the EtOH:water extract solvent system at different harvesting ages. Table S3. VIP values of the major contributing compounds in the PLS model. Table S4. Maximum percent inhibition of α-glucosidase activity of the C. caudatus E:W (80:20) extract at different harvesting ages. Figure S1. Full spectrum of a representative J-resolved spectrum of an EtOH:water (80:20) C. caudatus extract from δ 0.0 to 8.0. Key to metabolites: 1 – quercetin derivatives; 2 – rutin; 3 - chlorogenic acid; 4 - proline; 5 - fatty acid; 6 - acetic acid; 7- catechin. Figure S2. (A) Full 1H NMR spectra of 6-, 8-, 10-, 12- and 14-week-old samples of C. caudatus. (B) Expanded 1H NMR spectra of the samples in the range of δ 5.5 to 8.5. Samples were dissolved in CH3OH-d4-KH2PO4 buffer in D2O (pH 6.0). Key to metabolites: 1 – quercetin derivatives; 2 - rutin; 3 - chlorogenic acid; 4 - proline; 5 - fatty acid; 6 - acetic acid; 7 – catechin. Figure S3. Positive ion mode MS/MS spectra of peaks 1 to 5. Peak 1, rutin; peak 2, quercetin 3-O-glucoside; peak 3, quercetin 3-O-xyloside; peak 4, quercetin 3-O-arabinofuranoside and peak 5, quercetin 3-O-rhamnoside. Figure S4. Relative quantification of metabolites in 6-, 8-, 10-, 12- and 14-week-old samples of C. caudatus at different harvesting ages. An * indicates significant differences (p < 0.05), ** indicates (p < 0.01) and *** indicate (p < 0.001) compared to the 6-week-old sample. Values are expressed as the mean ± standard deviation (n = 6). (DOCX 376 kb) [file 12906_2019_2655_MOESM1_ESM.docx]

**Supplementary files**

**Table S1.** Maximum percent inhibition of α-glucosidase activity of different EtOH:water (E:W) extracts of *C. caudatus*.

| E:W system  (Test conc: 0.2mg/mL) | α-glucosidase inhibitory activity,  Maximum percentage inhibition (%) |
| --- | --- |
|  |  |
| 100:0 | 78.03 ± 5.02^a^ |
| 80:20 | 88.60 ± 5.09^a^ |
| 60:40 | 82.61 ± 3.09^a^ |
| 40:60 | 14.98 ± 2.13^b^ |
| 20:80 | 18.73 ± 3.68^b^ |
| 0:100 | 12.16 ± 4.08^b^ |
| Quercetin  (Test conc: 0.25mg/mL) | 95.10 ± 1.30^a^ |

**Table S2.** Pearson’s correlation coefficient (r) between TPC and α-glucosidase activity of the EtOH:water extract solvent system at different harvesting ages.

|  | TPC vs α-glucosidase | |
| --- | --- | --- |
|  | EtOH:water systems | Harvesting ages |
| r value | 0.434 | 0.407 |
| p value | 0.05 | 0.05 |

**Table S3.** VIP values of the major contributing compounds in the PLS model.

| **EtOH:water system** | | | **Harvesting age** | | |
| --- | --- | --- | --- | --- | --- |
| Chemical shift (ppm) | VIP values | Compound | Chemical shift (ppm) | VIP values | Compound |
| 1.32 | 3.32777 | Fatty acid | 1.32 | 3.63743 | Fatty acid |
| 6.44 | 2.42052 | Quercetin derivatives | 6.44 | 2.23107 | Quercetin derivatives |
| 2.04 | 2.17439 | Proline | 3.52 | 1.51875 | Chlorogenic acid |
| 1.92 | 1.63974 | Acetic acid | 6.28 | 1.32646 | Quercetin derivatives |
| 6.28 | 1.62746 | Quercetin derivatives | 5.24 | 1.14128 | Quercetin derivatives |
| 3.52 | 1.57539 | Chlorogenic acid | 1.92 | 1.0878 | Acetic acid |
| 2.56 | 1.36821 | Catechin | 7.56 | 1.01214 | Quercetin derivatives |
| 7.02 | 1.31238 | Quercetin derivatives | 2.04 | 0.966446 | Proline |
| 7.32 | 1.22013 | Quercetin derivatives | 7.28 | 0.93576 | Quercetin derivatives |
| 7.28 | 1.13074 | Quercetin derivatives | 6.52 | 0.891371 | Rutin |
| 6.24 | 0.878476 | Quercetin derivatives | 7.02 | 0.87753 | Quercetin derivatives |
| 7.36 | 0.873287 | Quercetin derivatives | 5.28 | 0.794774 | Quercetin derivatives |
| 5.24 | 0.848487 | Quercetin derivatives | 7.36 | 0.731015 | Quercetin derivatives |
| 6.52 | 0.792846 | Rutin | 6.24 | 0.71236 | Quercetin derivatives |
| 7.56 | 0.719848 | Quercetin derivatives | 2.56 | 0.705845 | Catechin |

**Table S4**. Maximum percent inhibition of α-glucosidase activity of the *C. caudatus* E:W (80:20) extract at different harvesting ages.

| E:W system  (Test conc: 0.2mg/mL) | α-glucosidase inhibitory activity,  Maximum percent inhibition (%) |
| --- | --- |
|  |  |
| 100:0 | 78.03 ± 5.02^a^ |
| 80:20 | 88.60 ± 5.09^a^ |
| 60:40 | 82.61 ± 3.09^a^ |
| 40:60 | 14.98 ± 2.13^b^ |
| 20:80 | 18.73 ± 3.68^b^ |
| 0:100 | 12.16 ± 4.08^b^ |
| Quercetin  (Test conc: 0.25mg/mL) | 95.10 ± 1.30^a^ |


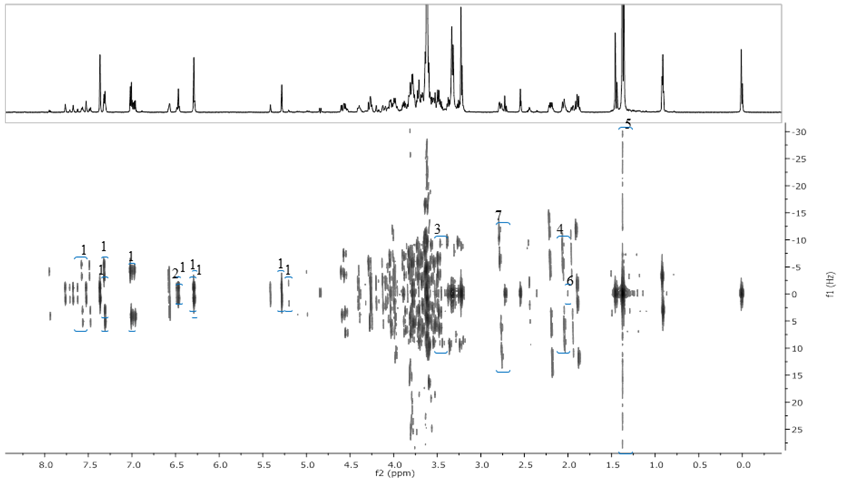


**Figure S1.** Full spectrum of a representative *J*-resolved spectrum of an EtOH:water (80:20) *C. caudatus* extract from δ 0.0 to 8.0. Key to metabolites: 1 – quercetin derivatives; 2 – rutin; 3 - chlorogenic acid; 4 - proline; 5 - fatty acid; 6 - acetic acid; 7- catechin.


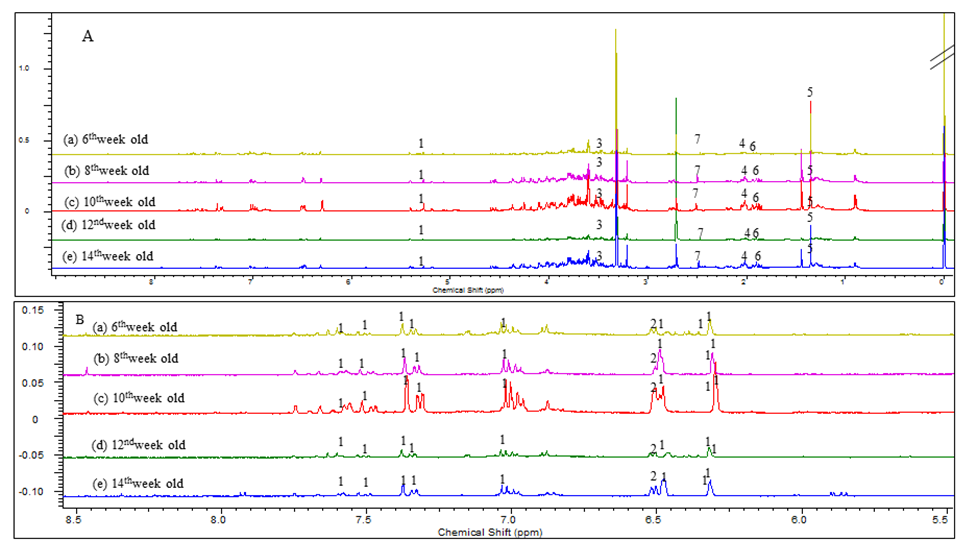


**Figure S2.** (A) Full ^1^H NMR spectra of 6-, 8-, 10-, 12- and 14-week-old samples of *C. caudatus*. (B) Expanded ^1^H NMR spectra of the samples in the range of δ 5.5 to 8.5. Samples were dissolved in CH_3_OH-*d*4-KH_2_PO_4_ buffer in D_2_O (pH 6.0). Key to metabolites: 1 – quercetin derivatives; 2 - rutin; 3 - chlorogenic acid; 4 - proline; 5 - fatty acid; 6 - acetic acid; 7 – catechin.


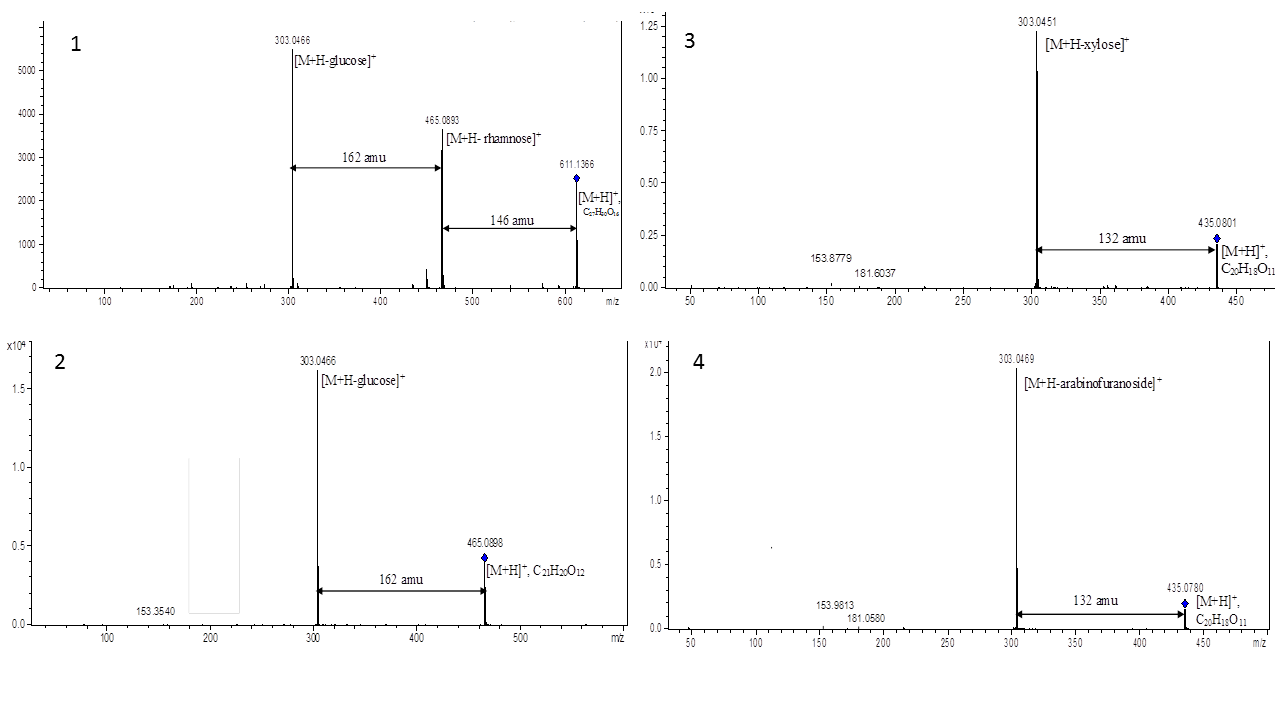


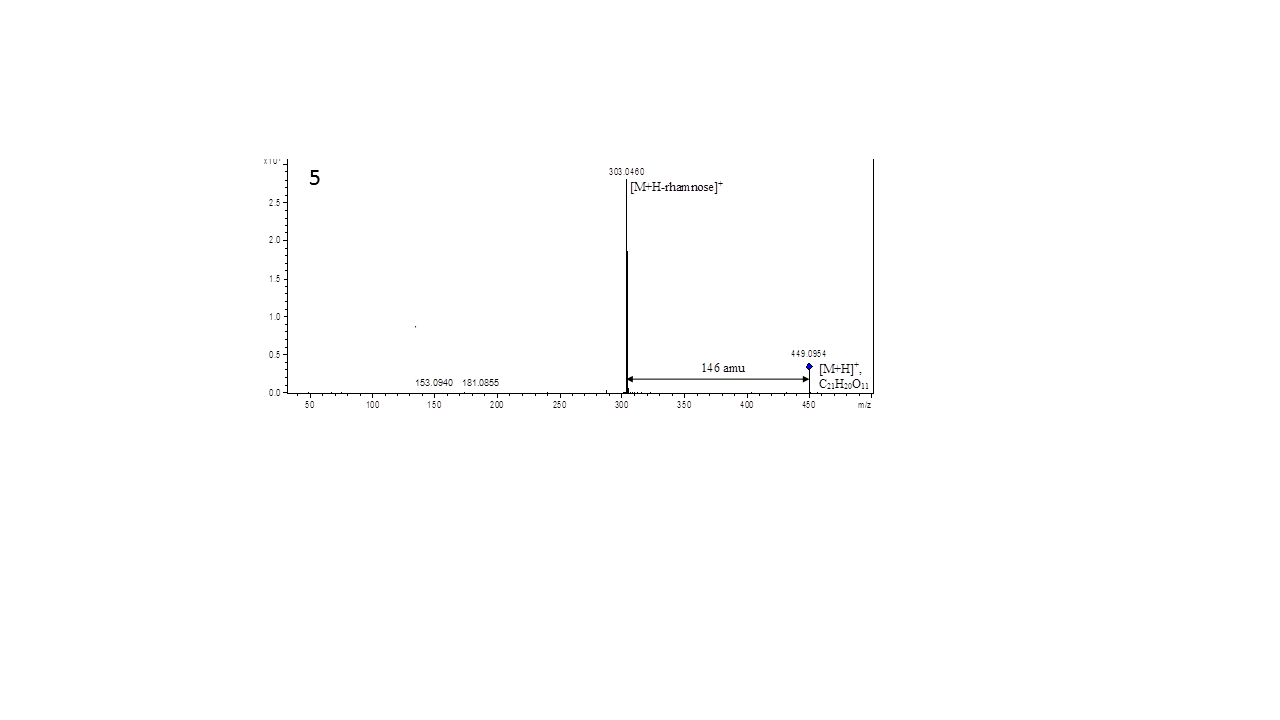


**Figure S3.** Positive ion mode MS/MS spectra of peaks 1 to 5. Peak 1, rutin; peak 2, quercetin 3-*O*-glucoside; peak 3, quercetin 3-*O*-xyloside; peak 4, quercetin 3-*O*-arabinofuranoside and peak 5, quercetin 3-*O*-rhamnoside.

**
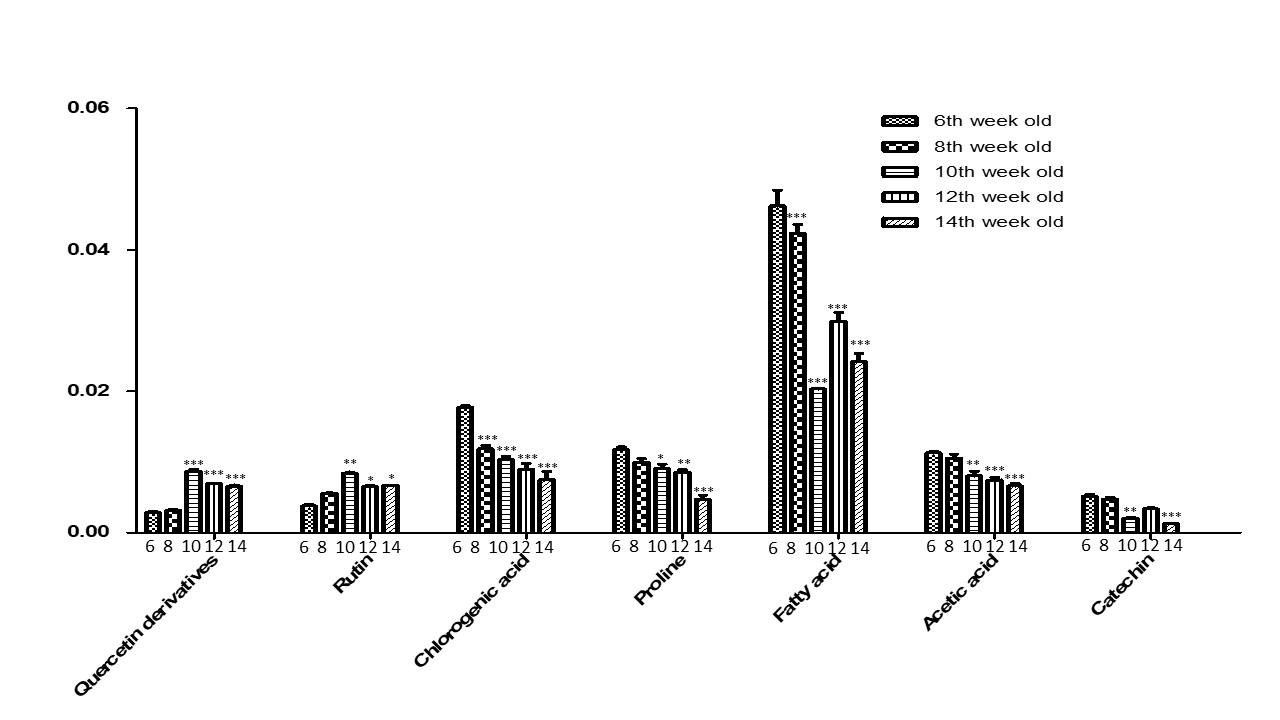
**

**Figure S4.** Relative quantification of metabolites in 6-, 8-, 10-, 12- and 14-week-old samples of *C. caudatus* at different harvesting ages. An * indicates significant differences (p<0.05), ** indicates (p<0.01) and *** indicate (p<0.001) compared to the 6-week-old sample. Values are expressed as the mean ± standard deviation (n=6).
